# Polytreatment in a very old patient. What’s better for safe prescribing?

**DOI:** 10.1002/alz.089856

**Published:** 2025-01-09

**Authors:** Pietro Gareri, Ilaria Gareri, Luciana Barbara Covello, Manuela Ammendola

**Affiliations:** ^1^ Department of Frailty ‐ Center for Cognitive Disorders and Dementia, Catanzaro Lido, ASP Catanzaro, Catanzaro Italy; ^2^ Center for Cognitive Impairment and Dementia, Catanzaro Italy; ^3^ School of Geriatrics ‐ University Magna Graecia‐Resident in training, Catanzaro Italy; ^4^ Mesoraca ‐ ASP Kroton, Kroton Italy; ^5^ Family Nurse ‐ ASL Mantova, Mantova Italy

## Abstract

**Background:**

Prescription for inappropriate drugs can be dangerous to very old people, due to the increased risk of adverse drug reactions.

**Case report:**

We report the consequences of inappropriate prescriptions in a 99‐year‐old woman. She had a clinical history of vascular dementia, diabetes, hypothyroidism, heart failure, osteoarthritis, chronic renal failure, and hypoacusia, and was admitted to our attention for asthenia and loss of appetite. She was hospitalized two months before for third‐degree atrioventricular block, and underwent pacemaker implantation. She had a moderate cognitive impairment, and functional dependence. Pharmacological history was collected by nurses through a video‐call and revealed that she was taking bisoprolol, furosemide, acetylsalicylic acid, L‐thyroxine, valsartan, gliclazide, folate, and digoxin (0.0625 mg/day). She had been suffering from nausea and loss of appetite for one month. On geriatric consultation, she had a heart frequency of 60 bpm. Vesicular murmur was normal, the abdomen was soft on palpation, and peripheral pulses were normal. Blood pressure was 130/80 mm Hg. Electrocardiography confirmed the presence of pacemaker rhythm. Echocardiography documented a mildly depressed ejection fraction of 45%. In the suspicion of digoxin intoxication, digoxin was soon interrupted. She started citicoline 1 g intramuscularly for the first ten days and then 1 g orally. Blood exams showed renal failure (ClCR 21.4 ml/min/m2), mild anemia, digoxin 2.7 ng/ml. A couple of days after digoxin interruption she started to feed again and felt much better.

**Conclusions:**

The present case report shows the need for wise prescriptions in very old people. Drug‐induced symptoms are the second most frequent medical problem that geriatricians encounter and collaboration with nurses can help to optimize pharmacotherapy by deprescribing inappropriate agents.

